# The Russian subjective and objective uncertainty stress (SOUS-14) scale: factor validity, internal reliability, and measurement invariance in university student sample

**DOI:** 10.3389/fpsyg.2025.1583172

**Published:** 2025-10-30

**Authors:** Yuri P. Zinchenko, Varvara I. Morosanova, Anna M. Potanina

**Affiliations:** ^1^The Federal State Budget Scientific Institution “Federal Scientific Center for Psychological and Multidisciplinary Research”, Moscow, Russia; ^2^Faculty of Psychology, Lomonosov Moscow State University, Moscow, Russia

**Keywords:** uncertainty stress, subjective uncertainty stress, objective uncertainty stress, scale validation, measurement invariance, university students

## Abstract

The rapid and non-predictable changes in various spheres of human and social life in the 21^st^ century have shifted research focus to the perceived uncertainty stress. Although observations show that the uncertainty stress exists and influences individuals in Russia, no significant attention has been paid to how we can measure it. Previous study presented the instrument for measuring uncertainty stress—a Russian “Subjective and Objective Uncertainty Stress (SOUS-14)” scale. The present study continues the work on validating this scale on different samples. It aims to examine SOUS-14 psychometric characteristics in university students, as well as to check its measurement invariance across gender groups and assess differences between them. The sample consisted of 621 Russian university students (mean age 19.09; 47.02% young women). The results confirmed high validity and reliability of the scale as well as its second-order factor structure. The study revealed that uncertainty stress construct is invariant across genders. It has been also shown that female students experience higher levels of uncertainty stress, than male students. Thus, the study confirmed that the developed scale has adequate statistical and psychometric characteristics, is applicable for studying uncertainty stress and its gender aspects, and can be used for research purposes.

## 1 Introduction

The problem of uncertainty as a cause of physiological and psychological stress is a well-developed field in psychology (e.g., Greco and Roger, 2003; Peters et al., 2017). Traditionally, uncertainty was associated with decision-making models and was understood as the presence of more than one alternative when the results of choosing one or the other are predicted probabilistically (Solntseva and Smolyan, 2009). Yet, in the 21st century the rapid and non-predictable changes in various spheres of human and social life have shifted researchers’ attention to the analysis of not only decision-making in uncertain situations, and somatic symptoms caused by them, but also subjective assessments of their perception, experience and evaluation as stressful (Fernández-García et al., 2024; McCarty et al., 2023; Sischka et al., 2024; Zolotareva, 2023), i.e., to the study of perceived stress. For example, during the COVID-19 pandemic, the situation was aggravated by fears for life and health and the inconsistency of huge flows of information about the disease, its treatment and prevention (Stankovska et al., 2020; Santomauro et al., 2021; McCarty et al., 2023; Kornienko and Rudnova, 2023). It is no coincidence that the experts called this period the “pandemic of psychological uncertainty stress” (Sweeny et al., 2020).

Experiencing uncertainty stress might lead to severe consequences. Empirical research has linked the perception of uncertainty to decreased mental health (Phillimore and Cheung, 2021) and increased distress (Massazza et al., 2023). It has been shown that uncertainty stress reduces the ability to effectively cope with various life events, negatively affects self-esteem (Peng et al., 2021), and contributes to the development of negative emotional states (Wise et al., 2023). This problem is especially acute in late adolescence and young adulthood, as young individuals during their transition between life stages are engaged in professional self-determination and career exploration—a highly uncertain process due to rapid changes in occupational world (Othman et al., 2019; Morosanova et al., 2024). A large number of recent cross-section and longitudinal studies report an increase in the level of anxiety and distress in college and university students across different countries and regions (Knapstad et al., 2021; Zhai and Du, 2024; Zarowski et al., 2024; Brown and Papp, 2024; Brown et al., 2024) due to demographic and socio-economic aspects (Ahamed and Limbu, 2024), digital fatigue (Krishna and Rajan, 2025), ecological factors (Clayton, 2020; Zinchenko, 2021; Hamaideh et al., 2022). Evidence support that perceived uncertainty and intolerance to uncertainty are important predictors of the recent increase in stress symptoms and decrease in students’ well-being (Satici et al., 2022; Ben Salah et al., 2023). Thus, the data indicate the need to study not only the perceived stress in students, but also the stress associated with their perception of various life situations, including highly uncertain situations.

In this connection, it is important to distinguish between subjective and objective uncertainty stress.

Subjective uncertainty stress occurs when an individual is placed in a difficult ambiguous situation provoking negative stressful experiences and a decrease in psychological well-being. This type of uncertainty becomes a stressor when an individual’s resources (i.e., cognitive, personal, and regulatory competencies) are insufficient for overcoming it and it takes time to actualize or develop those resources (Hobfoll, 2011). This type of stress is well-researched in the studies of perceived stress and intolerance to uncertainty and their influence on various life outcomes (e.g., Arbona et al., 2021; Sultanova et al., 2021). Subjective uncertainty concerns individuals’ problem situations such as dissatisfaction with relationships, school and work difficulties, lack of support from family members (Chernousova, 2022).

Contrary, objective uncertainty stress is much less studied. It arises in response to a subject’s encounter with not just new global challenges, but also objectively unpredictable ones such as natural disasters, pandemics, revolutions, and wars. According to cognitive appraisal (Lazarus and Folkman, 1984) and conservation of resources theories (Hobfoll, 2011), situations of objective uncertainty are always stressors, since their main characteristic is the objective lack of information about their nature, dynamics, and consequences. One of the key characteristics of objective uncertainty stress is that individuals cannot fully cope with it using resources they possess (Kriger, 2014), only mitigate their impact by accumulating psychological and external resources. This is especially relevant for adolescents and young adults, as during the above-mention sensitive period of career exploration it is extremely important to be able to (a) assess how destructive objective uncertainty stress can be during this period; (b) develop support measures to reduce its impact.

Thus, a person’s experience of uncertainty stress is associated with the following aspects: (1) assessment of the situation as tense/stressful; (2) the type of uncertainty of the given situation; (3) presence/absence of resources for overcoming stress.

An important aspect of perceived stress in young adults (and uncertainty stress in particular) is gender differences. Current literature on this issue suggests that young adult females tend to experience overall higher levels of stress than young adult males (Graves et al., 2021). Recent findings on stress, anxiety and negative emotional states during COVID-19 pandemic confirm this trend: young adult females tend to demonstrate higher levels of stress, anxiety, emotional distress and depression than males (Prowse et al., 2021; Rodriguez-Besteiro et al., 2021; Zarowski et al., 2024). Though there was some evidence from Chinese college students that gender had no significant effects on stress symptoms, such as anxiety, during the pandemic (Cao et al., 2020), majority of contemporary researchers report gender differences in perceived stress and stress symptoms. As for gender differences in uncertainty stress, recent work by T. Yang and colleagues showed, that gender was not significant predictor of the uncertainty stress (Yang et al., 2025). Meanwhile, literature analysis shows little to no data on gender differences in specifically subjective and objective uncertainty stress, since these types of stress have not been thoroughly studied yet.

The resent research calls for re-evaluation of previously developed stress assessment instruments (i.e., Rao et al., 2024; Muysewinkel et al., 2024), as well as creation of the new ones (Olasina, 2023). However, most of the new tools were developed during COVID-19 pandemic and cannot be used for evaluating uncertainty stress in more broad contexts. At the same time, despite the wide range of available methods for stress assessment, researchers note that quite many of them have not been thoroughly adapted and validated for student samples (e.g., Fernández-García et al., 2024). To address these issues, we have developed a new instrument—“Subjective and Objective Uncertainty Stress (SOUS-14),” presented in the article “Diagnostics of Stress of Subjective and Objective Uncertainty: Development and Validation of a Questionnaire” (Morosanova et al., 2024). This previous work was focused primarily on developing the concept of subjective and objective uncertainty stress and the instrument for its measurement. It also examined the differences in uncertainty stress between students who chose different educational tracks: college vs. university. Since we found significant differences in the level of uncertainty stress between university and college students, and due to the small sample of university students, the initial validation of the scale was carried out exclusively on a sample of college students.

The present study redresses these limitations and aims at: (1) psychometric evaluation of “Subjective and Objective Uncertainty Stress (SOUS-14)” scale on a large sample of Russian university students; (2) analysis of its measurement invariance and the gender sensitivity in terms of subjective and objective uncertainty stress.

## 2 Methods

### 2.1 Sample

The study involved undergraduate students aged 17 to 25 years attending state universities in Central (Moscow, Kaluga) region of Russia. Data was collected between October 2024 and January 2025. The questionnaires were administered via online “Testograf” platform.^1^ Using online administration has many advantages, primarily related to reducing the costs of study and the possibility to speed up data collection. However, these types of studies are susceptible to a number of selection biases associated with non-probability selection of respondents, such as “self-selection” (De Man et al., 2021), low response rate (Wu et al., 2022), etc. Although in our study we used the non-probability sampling method (conditional sampling), possible selection biases were controlled during the questionnaire administration procedure, which included: (1) providing links to the online-questionnaire in a controlled environment (classrooms in the presence of a lecturer); (2) eliminating the possibility of repeated completion by the same respondent; (3) including of a larger number of students from STEM specialties in the sample to equalize the number of young women and men (since the gender ratio in the social and humanitarian fields is often shifted toward a larger number of women). Participation in the study was encouraged by benefits during the post-term exams (additional points, fewer questions on the exam, etc.). Access to the questionnaires was provided individually to each student through a direct link to the project page, the transition could be made from any device available to the participants (smartphone, tablet, and laptop). The time for completion was limited to one academic hour, since the study implemented short and screening versions of the scales.

Statistical methods for bias control, such as weighting adjustments, were not used in this study. Regarding the minimum sample size, we did not perform a statistical power analysis, but were guided by the rule of “20 observations per measured variable/item,” which in our case (14 items) gave a minimum number of observations—280.

This allowed to obtain responses from 856 students, 229 of which were discarded due to incompletion. The final sample consisted of 621 students majoring in social sciences (35.10%) and STEM (64.90%). Mean age—19.09 ± 1.10; 47.02%—young women; 28.34%—freshmen, 55.52%—sophomore, 14.33%—junior and 0.81%—senior.

### 2.2 Instruments

1. A Russian “Subjective and Objective Uncertainty Stress—SOUS-14” scale (Morosanova et al., 2024). The SOUS-14 was developed based on the analysis of the existing life stress and uncertainty scales from T. Yang’s “The Student Daily Stress Questionnaire” (Yang et al., 2019) and perceived stress questionnaires. The initial pool of statements was formed on the basis of the existing Russian translation of the SDSQ (Banshchikova et al., 2023) and the Perceived Stress Scale adapted in Russian (Zolotareva, 2023). Four Russian researchers (PhD in Psychology) with experience in the field of stress psychology assessed the face and content validity of the initial set of statements. In addition, the items were checked by a philologist for compliance with Russian language norms. The scale was translated into English by a bilingual specialist with a degree in philology and psychology.

The SOUS-14 consists of 14 items related to situations that can cause stress reactions (*ω* in the initial validation study = 0.887): 7 of them for subjective uncertainty stress (initial ω = 0.832), and another 7—for the objective uncertainty stress (initial ω = 0.833). The respondents are asked to rate the degree of perceived stress in described situations on a 4-point scale where 1 is “no stress” and 4 is “excessive stress.” The integrative scale assesses the general level of uncertainty stress by summing up the scores obtained on the two scales: high score on this scale stands for severe uncertainty stress, and low score stands for absence of stress. The item examples include: “Отсутствие поддержки от членов семьи /Lack of support from family members” for subjective uncertainty stress, and “Климатические изменения и природные катаклизмы/Climate change and natural disasters” for objective uncertainty stress.

The following methods were used to compare SOUS-14 construct validity in university students with the previously obtained data (Morosanova et al., 2024):

1. V. I. Morosanova’s “Self-Regulation Profile Questionnaire—SRPQM” (Morosanova and Kondratyuk, 2020). The SRPQM is based on the resource approach to conscious self-regulation (SR) by Morosanova (2021). Within this approach, SR is considered a meta-resource for achieving life goals that ensures coordinated interaction of all individual resources (motivation, temperament, emotional processes, etc.). The instrument assesses the general ability for conscious self-regulation and its components, consistently manifested in various types of the human voluntary activity and life situations, i.e., the regulatory resources of a person for achieving their goals. The questionnaire consists of 28 items and 7 subscales: “Planning” (PL), “Modeling” (M), “Programming” (PR), “Results evaluation” (RE), “Flexibility” (F), “Independence” (I), “Reliability” (R) as well as the integrative scale “General Level of Self-Regulation” (GLSR) calculated as the sum of scores on all scales. The respondents are asked to rate their agreement with the statements about their regulatory features on a 5-point scale where 1 is “wrong” and 5 is “right.”
2. “Perceived Stress Scale, PSS-10” (Cohen et al., 1983, Russian adaptation by Zolotareva, 2023) consisted of 10 items and 2 subscales “Distress” and “Coping” as well as an integrative scale of the general level of perceived stress (sum of points). The respondents are asked to rate how often they felt psychological discomfort and their coping abilities during last month on a 5-point scale where 1 is “never” and 5 is “often.”

Socio-demographic data, such as income level and living conditions were not collected in this study.

### 2.3 Statistical analysis

Data analysis was performed using JASP ver. 0.18.3.0. Statistical procedures included descriptive statistics, Pearson’s correlation analysis, confirmatory factor analysis (CFA), McDonald’s omega and independent samples t-test. Correlation analysis, McDonald’s omega and confirmatory factor analysis (including multigroup CFA for measurement invariance analysis) were used to check for invariance of SOUS’ psychometric properties on a different sample. CFA was also used to examine the measurement invariance across gender groups. The goodness-of-fit of the models in CFA was estimated using the following fit indices: chi-square (χ^2^), degrees of freedom (df), comparative fit index (CFI), Tucker–Lewis index (TLI), standardized root mean square (SRMR), and root mean square error of approximation (RMSEA). Good model fit was defined by CFI ≥ 0.95, TLI ≥ 0.95, RMSEA ≤ 0.06, SRMR ≤ 0.06. Measurement invariance was analyzed by comparing three models reflecting three types of invariances (configural, metric, and scalar) by calculating *Δ* CFI, Δ RMSEA, Δχ^2^ and Δdf (Van De Schoot et al., 2015). All types of invariances were evaluated with diagonally weighted least squares (DWLS) estimator. Finally, independent samples t-test was performed to examine gender differences in SOUS scale scores.

## 3 Results

### 3.1 Descriptive statistics and correlations

Table 1 shows descriptive statistics and correlations for all indicators. According to the results, university students experience low to middle levels of uncertainty stress. The distribution is close to the normal for all variables (skewness and kurtosis values do not exceed 1 in absolute value). For this reason, the parametric methods of analysis were further applied. The correlation analysis demonstrated results similar to our previous work: low to moderate significant correlations between SOUS-14 scales and the levels of conscious self-regulation and perceived stress. In particular, the general level of uncertainty stress moderately positively correlated with general level of perceived stress and “Distress.” Thus, the SOUS-14 questionnaire demonstrates good indicators of construct validity on the sample of university students. The results also show weak negative correlation with general level of conscious self-regulation.

**Table 1:** Descriptive statistics, Shapiro–Wilk test and correlations for all indicators (*n* = 621).

Indicators	M(SD)	Sk	Kr	1	2	3	4	5	6
1. Subjective uncertainty stress	13.95(4.55)	0,35	−0,75	–					
2. Objective uncertainty stress	12.65(3.74)	0,35	−0,70	0.58***	–				
3. General level of uncertainty stress	26.60(7.31)	0,30	−0,63	0.91***	0.86***	–			
4. General level of self-regulation	93.33(15.05)	0,06	−0,36	−0.15***	−0.25***	−0.22***	–		
5. Distress	16.07(4.96)	−0,13	−0,41	0.35***	0.49***	0.46***	−0.38***	–	
6. General level of perceived stress	25.25(6.70)	−0,02	−0,42	0.30***	0.45***	0.41***	−0.51***	0.90***	–

**p < 0.01; ***p < 0.001; Sk, Skewness; Kr, Kurtosis.

### 3.2 Confirmatory factor analysis and internal consistency

According to our previous work, we tested for only one model: with two first-order factors and one second-order factor (subjective and objective uncertainty stress scales and general uncertainty stress scale). Since the data demonstrate a low deviation from normality, and there was a fairly large sample, the unweighted least squares method was used to assess the model fit. The factor loading threshold was 0.4.

**Figure 1:**
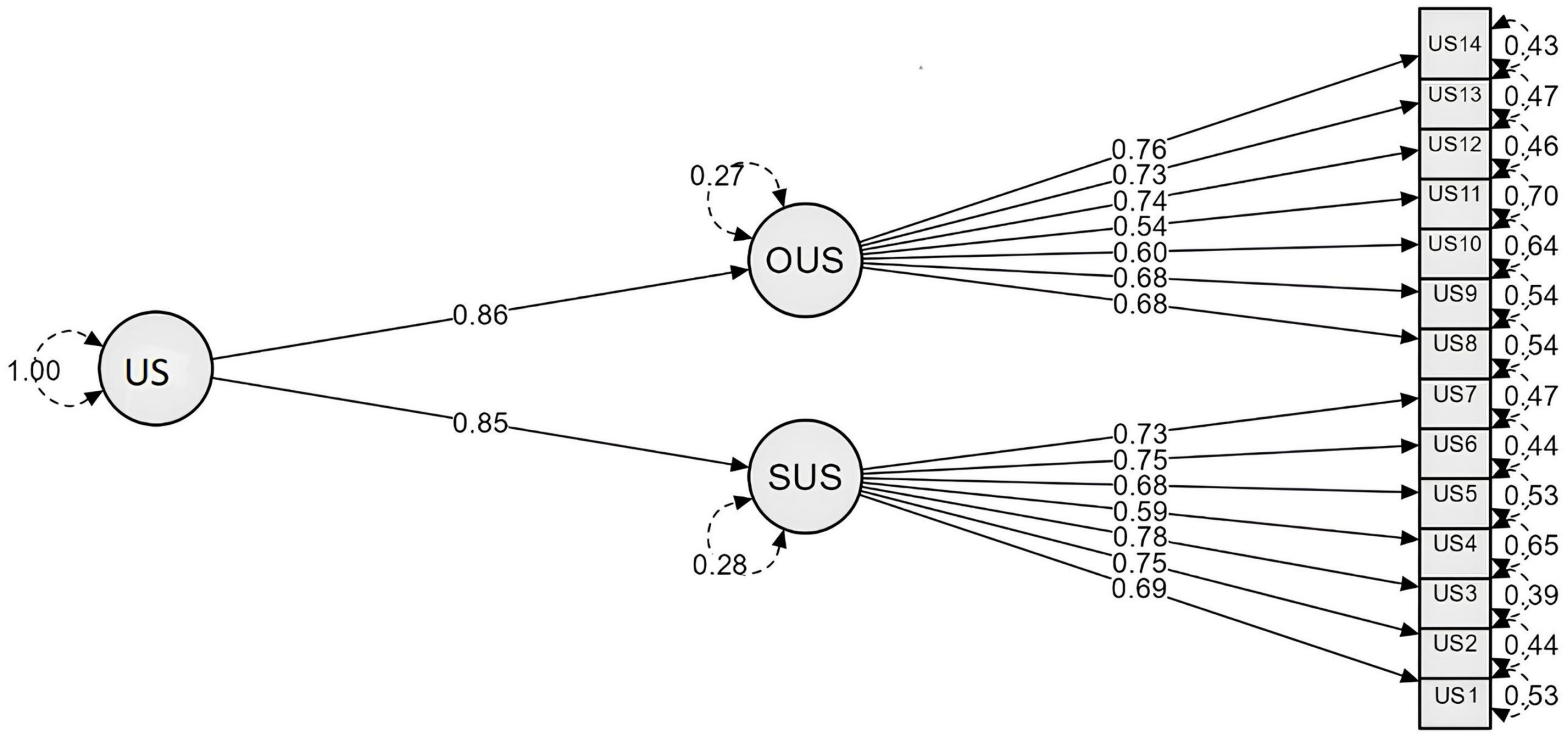
SOUS-14 CFA model.

According to the data obtained, the model showed good fit indices: χ^2^ = 254.64, *df* = 75, CFI = 0.982, TLI = 0.971, RMSEA = 0.062, SRMR = 0.063. The fit indices of this model are not very different from the final model verified in our previous study: *Δ* CFI = 0.006; Δ TLI = 0.015; Δ RMSEA = 0.006; Δ SRMR = 0.005 (Morosanova et al., 2024). The range of factor loading coefficients in current CFA model were from 0.54 to 0.78. Figure 1 shows the factor loadings of the items on the scales as well as the contribution of the common factor to explaining the covariance between the first-order latent factors.

McDonald’s Omega coefficient was calculated to evaluate internal consistency of SOUS-14 scales. According to the obtained results, all three scales demonstrate high internal consistency in university students. For subjective uncertainty stress: *ω* = 0.838; for objective uncertainty stress: ω = 0.816; for the general level of uncertainty stress: ω = 0.878.

### 3.3 Gender differences and measurement invariance across genders

First, we examined measurement invariance across genders to assess the applicability of SOUS-14 in male and female groups. Since in this type of analysis we compared nested models in smaller groups (*n* = 292 for male and *n* = 329 for female), for multigroup CFA we chose diagonally weighted least squares (DWLS) estimator. Initially we tested the configural invariance, i.e., assessed our model for each gender group. Next, we analyzed metric invariance, i.e., tested if the factor loadings were equal across gender groups (Van De Schoot et al., 2015). Finally, we assessed scalar invariance by constraining factor loadings and intercepts to be equal. Table 2 shows the results of baseline and difference tests of three models corresponding to each type of invariance. The results revealed that all baseline models showed acceptable fit indices (CFI ≥ 0.95; RMSEA ≤ 0.06). Difference test between configural invariance model and metric invariance model showed no significant differences both in the Δχ^2^ (*p* = 0.545) and in fit indices (ΔCFI = 0.001; ΔRMSEA = 0.002). Thus, our data shows the invariance of factor loadings between gender groups. Difference test between metric invariance model and scalar invariance model revealed significant differences in the Δχ^2^ (*p* < 0.001), indicating that null hypothesis about equal loadings and intercepts should be rejected. However, otherwise the model for scalar invariance showed good fit to the data (ΔCFI = 0.002; ΔRMSEA = 0.000). Since informational criteria are considered superior to Δχ^2^ due to their independence to sample size (Meade et al., 2008), we made the final decision about the hypothesis based on ΔCFI and ΔRMSEA. According to observed changes in fit indices, our data shows scalar invariance in gender groups. Thus, the SOUS-14 scale is applicable for measuring gender differences in uncertainty stress.

**Table 2:** Model fit indices for measurement invariance across gender groups.

Model	CFI	RMSEA	Baseline test			Difference test				
			χ^2^	df	*p*	Δ CFI	Δ RMSEA	Δχ^2^	Δdf	*p*
Configural invariance	0.982	0.065	343.39	150	<0.001					
Metric invariance	0.981	0.063	363.36	163	<0.001	0.001	0.002	19.97	13	0.545
Scalar invariance	0.979	0.063	415.93	188	<0.001	0.002	0.000	52.57	25	0.019

Next, we ran independent samples t-test to examine gender differences between SOUS-14 values. Table 3 shows the test results: female students demonstrated significantly higher subjective, objective and general uncertainty stress than male students. The effect size was medium for all variables (Cohen’s d *ϵ* [0.5–0.6]).

**Table 3:** Gender differences for SUS, OUS and US total scores.

SOUS scales	Female (*N* = 292)	Male (*N* = 329)	Welch t	Cohen’s d [95% CI]
	M(SD)	M(SD)		
Subjective uncertainty stress	15.16 (4.77)	12.87 (4.05)	6,42***	0.519 [0.358; 0.679]
Objective uncertainty stress	13.95 (3.71)	11.50 (3.39)	8,55***	0.689 [0.526; 0.851]
General level of uncertainty stress	29.11 (7.49)	24.37 (6.51)	8,37***	0.676 [0.513; 0.838]

Effect size is given by the Cohen’s d; ***p < 0.001. Since Brown-Forsythe test suggested violation of the equal variance assumption, Welch t-test was applied.

## 4 Discussion

The study examined and reported the psychometric characteristics of SOUS-14 scale among university students in Russia. It also assessed measurement invariance across gender groups and gender differences in uncertainty stress in male and female students.

First, our results confirmed the validity of SOUS-14 in university students. Positive moderate correlations of the SOUS-14 scales with general level of perceived stress and distress level correspond both with the data on the relationship between uncertainty and perceived stress (Wu et al., 2021; Reizer et al., 2021) and our previous findings on college students (Morosanova et al., 2024). Moderate size of correlation coefficients confirms that uncertainty stress and perceived stress are conceptually close, but distinct constructs. Thus, SOUS-14 might supplement data obtained with PSS-10 with a detailed information about the severity of different types of uncertainty stress.

Interestingly, subjective uncertainty stress showed weaker connections with perceived stress in university students compared to college students (Morosanova et al., 2024). Note, that these results still do not fully explain differences in uncertainty stress between university and college students, particularly whether it is due to specifics of these educational tracks in Russia. Regional specifics might also explain these differences. University students in our study were from the same geographical region, and college sample included students from different Russian regions (e.g., Far East). Resent findings by T. Yang and his colleagues support this assumption: uncertainty stress shows high geographical variation (Yang et al., 2025). This issue should be investigated further in the future studies.

Negative correlations found between the questionnaire scales and the general level of conscious self-regulation are consistent with our data on the negative association between the development of SR and stress (Morosanova, 2021; Kondratyuk and Morosanova, 2021). This is consistent with theoretical and empirical evidence for self-regulation preventing stress development and mitigating its effect on psychological well-being and academic success in adolescents and young adults (Morosanova, 2021; Barbayannis et al., 2022). Recent work on the Chinese sample also supports this finding: self-regulatory fatigue found to strengthen the relationship between intolerance to uncertainty and academic burnout (Qiang et al., 2024). Additionally, our findings suggest that self-regulation might act as a universal resource for coping with uncertainty stress. Previous studies on the Russian sample during the COVID-19 pandemic showed that successful coping with uncertainty was associated with components of conscious self-regulation (Zinchenko et al., 2020). Testing this assumption in different contexts might be one of the future research directions.

Second, the reliability analysis demonstrated high internal consistency of SOUS-14 in the sample of university students—McDonald’s Omega were above 0.8 for all scales. This is consistent with both the reliability data of original Yang’s questionnaire (Yang et al., 2019) and the internal consistency of SOUS-14 scale in college students (Morosanova et al., 2024). The confirmatory factor analysis supported the suggested second-order factor structure, proposed and validated on the college students’ sample (Morosanova et al., 2024). Thus, our questionnaire allows for measuring uncertainty stress with a high degree of reliability on different student samples.

Third, our results indicate that the model describing SOUS is not gender-biased and this instrument is applicable for studying gender differences. Thus, the study revealed that female students have significantly higher levels of both subjective and objective uncertainty stress, than male students. This finding corresponds with existing literature on gender differences in perceived stress in general (Graves et al., 2021), and the data on stress in young adult females obtained in situations of uncertainty (like COVID-19; Prowse et al., 2021; Clabaugh et al., 2021). However, our results differ from those of Yang et al.: they found no significant contribution of gender to uncertainty stress (Yang et al., 2025). Moreover, their result showed higher life stress in male students, while we found higher subjective uncertainty stress (which is conceptually close to life stress) in female students. We suggest this might be due to different methodology behind SOUS-14 and SDSQ. According to resources theories of stress underlying SOUS-14, it is the depletion (or lack of) of psychological resources that causes higher stress, not the type of stressful situation itself. Another explanation might be the above-mentioned geographical variation and cross-cultural differences. Global meta-analysis on the negative emotions during COVID-19 pandemic reported higher increase in depression, anxiety and stress in European females compared to Asian and American (Daniali et al., 2023). Still, further research is needed for more in-depth understanding of the interplay between socio-economic, geographic, and psychological factors in subjective and objective uncertainty stress gender differences.

Thus, our study demonstrated that SOUS-14 is a reliable and valid tool for measuring the uncertainty stress in both college and university students. This instrument might also be used to assess gender differences in subjective and objective uncertainty stress levels. The SOUS-14 questionnaire has potential for the practical application. It opens up new possibilities in psychological and pedagogical practice for diagnosing the sources of increase in uncertainty stress in adolescents and young adults and providing them with counseling services. Yet, the study has certain limitations, since it does not address the issue of measurement invariance between different age groups. Besides, the SOUS-14 is still applicable only to the studies on student samples (17–25 years). Further research could be aimed at validating the questionnaire on samples of other ages, i.e., adults. Developing a version of the SOUS for younger adolescents and children could also have theoretical and practical significance. In addition, the study involved only students from large cities in the Central region of Russia, which raises the question of the questionnaire validity for the students living in other regions and/or in smaller cities. Another limitation is the lack of longitudinal validation of this questionnaire. Finally, the cross-cultural validity of the instrument has not yet been assessed. Future research will consider these limitations.

Another quite promising research direction is to study the impact of various aspects of uncertainty stress on academic success, subjective well-being, personal and professional development in students.

## Data Availability

The raw data supporting the conclusions of this article will be made available by the authors, without undue reservation.

## Footnotes

^1^ https://www.testograf.ru/

## Appendix

Instructions, statements, and key to the “Subjective and Objective Uncertainty Stress—SOUS-14”

Instructions:

You will be given some statements related to situations that can cause negative stress reactions. Please, evaluate the extent of possible stress in each of these situations on a scale: 1, no stress; 2, slight stress; 3, significant stress; 4, excessive stress.

Items:

1. Difficulties in studies
2. Unsatisfying relationships with peers
3. Conflicting relationships with teachers
4. Dissatisfaction with romantic relationships
5. Regular financial difficulties
6. Lack of support from family members
7. Threats to life and health
8. A rapidly changing world
9. Flow of negative news
10. In today’s world it is difficult to identify what is the truth and what is the lie
11. Climate change and natural disasters
12. Difficulties in projecting professional plans under conditions of uncertainty
13. It is difficult to understand who are the enemies and who are the friends
14. Growing uncertainty about the future

Key. The scales indicators are calculated by summing up the scores on the following items: subjective uncertainty stress—items 1–7; objective uncertainty stress—items 8–14. The indicator of the integral scale of the general level of uncertainty stress is calculated as the sum of the values on both scales of the questionnaire.
